# Spontaneous resolution of isolated neurogenic blepharoptosis after blunt trauma: A case report and literature review: Erratum

**DOI:** 10.1097/MD.0000000000013764

**Published:** 2018-12-14

In the article, “Spontaneous resolution of isolated neurogenic blepharoptosis after blunt trauma: A case report and literature review”,^[1]^ which appears in Volume 97, Issue 44 of *Medicine*, the authors would like to add “This research was supported by the National Research Foundation of Korea (NRF) funded by the Ministry of Science, ICT & Future Planning (2017R1C1B1006736).”
